# Biogenic CuO and ZnO Nanoparticles as Nanofertilizers for Sustainable Growth of *Amaranthus hybridus*

**DOI:** 10.3390/plants11202776

**Published:** 2022-10-19

**Authors:** Dali Vilma Francis, Neeru Sood, Trupti Gokhale

**Affiliations:** Department of Biotechnology, Birla Institute of Technology and Science, Dubai Campus, Dubai International Academic City, Dubai P.O. Box 345055, United Arab Emirates

**Keywords:** CuO nanoparticles, ZnO nanoparticles, hydroponics system, foliar application, *Amaranthus hybridus*

## Abstract

The biogenic synthesis of CuO and ZnO nanoparticles (NPs) was carried out by *Stenotrophomonas maltophilia.* The shape, size, and chemical identity of the CuO and ZnO NPs were determined using FTIR, XRD, SEM, EDX, and TEM analysis. The study aimed to investigate the effects of the CuO and ZnO NPs on *Amaranthus hybridus* seed germination and plant growth. Two different fertilizer application modes (hydroponics and foliar) were studied with varying concentrations of CuO (0.06 µM, 0.12 µM) and ZnO (0.12 µM, 0.24 µM) nanoparticles with water control and Hoagland’s media control. The hydroponic system of fertilizer application demonstrated better efficiency in terms of plant growth as compared to the foliar application. The agronomic traits, SPAD value, total reducing sugars, antioxidant activity, amount of copper, and zinc ions in root and shoot were analyzed for all experimental plants and found better with the nanoparticle application. The highlight of the study is the application of extremely low concentrations of CuO and ZnO nanoparticles, almost 70% lower than the copper and zinc salts in the Hoagland’s medium for improved plant growth. The use of lower concentrations of nanoparticles can prevent their accumulation in the environment and also lower the production cost. The high antioxidant concentration exhibited by the plants treated with CuO and ZnO nanoparticles ensures the enhanced plant’s resistance to infections and pests while promoting plant growth.

## 1. Introduction

The global population is expected to grow up to 9.7 billion by 2050 [1]. This rise in the global population will result in a 70 percent increased demand for food [2,3]. This massive challenge requires combined efforts to support intensive agriculture while preserving natural resources and reducing the adverse effects on the environment [4]. The extensive use of organic amendments and mineral fertilizers already created a detrimental impact on water and soil quality across the world [5]. Crop quality and productivity can be enhanced with the implementation of nutrition fertilization, while maintaining the soil fertility [6,7]. A customized nutritional requirement of crops, hence, needs to be catered by exploring non-disrupting technologies, improving the efficiency of agricultural systems, and implementing eco-friendly and solid agronomic practices [8,9,10].

Zinc and copper ions are vital elements for plant growth. Zinc is required for all six enzyme classes (oxidoreductases, hydrolases lyases, transferases, isomerases, and ligases) [11], as it aids in numerous metabolic processes and boosts the synthesis of chlorophyll, carotenoids, and the photosynthetic system of plants [12]. Copper is an active metal involved in plant redox reactions [13]. It is also a component of regulatory proteins, a cofactor of phenol oxidases and superoxide dismutase (SOD), and contributes to electron transport in the respiratory and photosynthetic chains [14]. Hence, it is important to improve the usage efficiency of copper and zinc as fertilizers [15].

Crop production is also immensely affected by infestation by insect pests or fungal, viral, and bacterial infections [16]. Similar to the use of chemical fertilizers, the application of traditional pesticides on plants also presents the issues of toxicity to humans and inadvertent environmental pollution [17]. Therefore, there is an immediate need for sustainable alternative techniques to enhance crop yield and manage plant pests and diseases.

A nanofertilizer may be defined as a material in the nanometer scale that contains micronutrient or macronutrients that can be delivered to the crops in a controlled mode [18,19,20]. Their higher surface area to volume ratio provides greater reactivity and deeper penetration into the plant, as well as soil [21]. Nanofertilizers are also required in small quantities, as they display gradual and sustained release of material, leading to enhanced plant productivity and efficient nutrient usage, further leading to a reduced environmental impact. Moreover, properties for targeted delivery can help leverage the potential of precision and sustainable agriculture [22,23]. Nanoparticles can be delivered in plants through foliar application and the hydroponic system. The hydroponic system is a practice of soilless agriculture that engages plant growth in liquid nutrient medium and is useful in vertical farming [9,24]. However, in foliar application, liquid nutrients are directly applied to the plant tissues in small amounts, thereby lowering the risk of accumulation in soil, while reducing the time for uptake as translocation from roots is not required [9]. The small size of nanofertilizers is envisaged to increase the area of contact with the leaf surface, thereby increasing the passage through the stomatal pore (diameter 1 µm) [9,25].

Few researchers studied the effect of copper and zinc nanoparticles on plant metabolism and vigor [26,27]. Abbasifar et al. (2020) reported an increase in chlorophyll content and antioxidant enzyme in basil plants treated with various concentrations of copper and zinc nanoparticles [28], while Rui et al., 2016 reported improved growth and chlorophyll content in peanut plants treated with FeO nanoparticles [29].

While CuO and ZnO nanoparticles enhance the plant growth, excessive amounts of the same may exhibit negative effects on the plant growth. Singh D and Kumar A (2016) reported the negative effect of increasing concentrations of CuO and ZnO nanoparticles on spinach. A concentration of ten milligrams per liter (mg/L) or less was shown to have no harmful effect on plants, with the metal absorption almost identical to the control. However, CuO and ZnO nanoparticle concentrations above 1000 mg/L reported a retarded growth of spinach plant. These studies conclude that the concentration of nanoparticles determines the positive or negative effect on the plants [30].

*Amaranthus hybridus*, a fast-growing, leafy vegetable that is favored for its extra nutritional content and flavor, is chosen for the study, as there are no reports on the effect of CuO and ZnO nanoparticles on its growth [31]. In the present study, biogenic synthesis of copper oxide (CuO) and zinc oxide (ZnO) nanoparticles was achieved using the soil bacterium, *Stenotrophomonas maltophilia* [32]. The effects of CuO and ZnO nanoparticles were tested on seed priming, plant growth, and phytochemical and antioxidant activity of *Amaranthus hybridus* under hydroponic and foliar application. It is envisaged that the influence of nanoparticles will promote germination rate and plant growth. An increased quality and quantity of yield is expected with minimal or no phytotoxicity. Since the nanoparticles are applied at significantly lower concentrations, they should not be accumulated in the plant tissue. The authors also compared the plant growth among hydroponics and foliar treatments to understand the effective mode of nanofertilizer administration. The objectives of this study were to determine the (1) optimum application concentration of CuO and ZnO nanoparticles; (2) effects on nanoparticles on seed germination; (3) administer the nanoparticles through the hydroponic and foliar system; (4) changes in phenotypic characters and pigments; (5) anti-oxidative enzymatic activity; and (6) uptake of copper and zinc in the plant. We anticipate the study will improve our understanding on the potential of nanoparticles as nanofertilizers in agriculture.

## 2. Materials and Methods

### 2.1. Synthesis of CuO and ZnO Nanoparticle

CuO and ZnO nanoparticles were synthesized in the laboratory using a bacterium, *Stenotrophomonas maltophilia*. The studies were carried out in 250 mL Erlenmeyer flasks, which contained 100 mL sterile Luria Bertani broth. The broth was inoculated with 1 mL of 18-hour-old culture of *S. maltophilia* (O.D 0.8 at 600 nm). The inoculated broth was incubated at 30 °C at 150 rpm, for 24 h to allow the culture to grow. CuSO_4_ or ZnSO_4_ stock solutions were added to two culture flasks, respectively, to attain a 2 mM concentration, while the pH of the media was adjusted to 8 using 1 M NaOH. The flasks were further incubated at 40 °C for three days at 200 rpm. After the incubation, the cells were lysed using a QSONICA sonicator with an amplitude of 40% and pulse of 15 s for 10 min under ice-cold conditions, and the cell lysate was centrifuged at 4000 rpm for 5 min. The supernatant was vacuum filtered through a 0.22 µm cellulose acetate membrane, and the filtrate was dialyzed using a snakeskin dialysis membrane (10 K MWCO). The dialyzed samples were lyophilized to obtain the powdered CuO and ZnO nanoparticles, respectively. The nanoparticles were characterized using UV–Visible spectroscopy, particle size analysis, X-ray diffraction, transmission and scanning electron microscopy analysis, and energy dispersive X-ray spectroscopy.

### 2.2. Seed Priming

*Amaranthus hybridus* seeds were received from Kerala Agricultural University, Mannuthy, India. Seeds were surface sterilized with 5% Sodium hypochlorite for 5 min and rinsed thoroughly with sterile DI water [33]. Next, 10 g of seeds were soaked for 8 h in 50 mL of 0.06, 0.12, and 0.24 µm CuO and ZnO nanoparticles solution, with sterile DI water as a negative control. Additionally, 1 mM ZnSO_4_ solution was maintained as a positive control, as it demonstrated a positive impact on seed priming [34]. After priming, the seeds were rinsed thoroughly with sterile DI water, dried, and a germination test was performed using the top of paper method [34]. The primed seeds were placed on three layers of sterile filter paper moistened with DI water, weighing approximately three times the dry weight of paper with 12 h light and 12 h dark periods at 15 °C. The germination percentage was calculated at 0, 18, 24, 36, and 48 h using the formula [34].

$$\mathrm{Germination}\;\%=\frac{\mathrm{Number}\;\mathrm{of}\;\mathrm{seedling}\;\mathrm{emerged}}{\mathrm{Total}\;\mathrm{number}\;\mathrm{of}\;\mathrm{seeds}\;\mathrm{planted}}\times 100$$

### 2.3. Composition of Modified Hoagland’s Medium [35]

Modified Hoagland’s medium was used for plant growth in the hydroponic system. The media composed of 6 mM KNO_3_, 4 mM Ca (NO_3_)_2_, 2 mM MgSO_4_, 1 mM NH_4_PO_4_, 46 μM H_3_BO_3_, 14.3 μM MnCl_2_, 0.8 μM ZnSO_4_, 0.4 μM CuSO_4_, 0.1 μM H_2_MoO_4_, and 44.4 μM Fe EDTA. The pH of the medium was adjusted to 6.5 using 2M KOH. CuSO_4_ and ZnSO_4_ salts were replaced by CuO and ZnO nanoparticles at varying concentrations in the study.

All the chemicals used in the study were of analytical grade and purchased from Sigma Aldrich chemical Co., St. Louis, MO, USA.

### 2.4. Selecting the Optimal Concentration of CuO and ZnO Nanoparticles for Plant Growth

The seeds germinated in DI water were transferred to rockwool placed in plastic seed trays, which were immersed in 500 mL of half-strength modified Hoagland nutrient solution. Three seedlings per tray were considered in triplicates for each study. CuSO_4_ and ZnSO_4_ salts in the medium were replaced with 0.012, 0.06, 0.12, 0.24, and 0.36 µm CuO and ZnO nanoparticles, respectively. A positive control was maintained in half strength Hoagland’s medium, while tap water was used as media control. Growth of the shoot was monitored to determine the rate of attaining the three-leaf stage. This growth rate was compared to determine the most efficient concentration of CuO and ZnO nanoparticles for plant growth.

### 2.5. Growth in Hydroponic System and Pot Study

The two concentrations of CuO and ZnO nanoparticles supporting optimal growth of *Amaranthus hybridus* till the three-leaf stage (as determined in the previous experiment) were selected for further studies. DI water-germinated seeds of *Amaranthus hybridus* were transferred to half-strength Hoagland’s medium for seven days. After attaining the three-leaf stage, half plants were transferred to the pot filled with commercial potting soil for foliar study while the rest were continued in the hydroponic system. A controlled environment with 4500–5000 lux, 12 h light/dark photoperiod, with relative humidity at 55%, was maintained for both systems. Media and negative controls were maintained for both systems.

#### 2.5.1. Foliar Application

Round plastic pots (size 20 × 30 cm) were filled with 300 g of commercial potting soil and watered till saturation using tap water. The three leaf plantlets of *Amaranthus hybridus* grown in half-strength Hoagland’s medium were transferred in individual pots. Four experimental setups were designed, ensuring the maintenance of triplicates for each treatment. All pots were watered with tap water at regular intervals. The CuO (0.06 and 0.12 μM) and ZnO (0.12 and 0.24 μM) nanoparticles were sprayed on the plant leaves through application of half-strength modified Hoagland’s medium, in which CuSO_4_ and ZnSO_4_ salts were replaced with CuO and ZnO nanoparticles, respectively. A media control was maintained by spraying half-strength modified Hoagland’s medium on the plant leaves, while a negative control was maintained with tap water. All plants were watered daily with 20 mL tap water, while the foliar application (through spraying) on the leaves for each experimental setup was performed twice a day.

All plants grown under the hydroponic and foliar setups were harvested on the 60th day and studied for their growth parameters, such as shoot length, root length, number of leaves, fresh weight, dry weight, and chlorophyll content. To determine the dry weight, harvested plants were dried at 40 °C till at a constant weight. The dried plants were pulverized and stored in airtight containers at 4 °C for further analysis.

#### 2.5.2. Hydroponic System

Four experimental setups were designed to study the effect of nanoparticle application on plant growth in hydroponic systems. Three seedlings per tray were considered in triplicates for each concentration of the nanoparticle under study. Plantlets grown till the three leaf stage were transferred to half-strength modified Hoagland’s medium, replacing CuSO_4_ salt with 0.06 and 0.12 μM CuO nanoparticles and ZnSO_4_ salt with 0.12 and 0.24 μM ZnO nanoparticles. The nutrient medium was changed every three days during the study. The plantlets grown in half-strength modified Hoagland’s medium were maintained as media control, while those grown in tap water were used as negative control.

### 2.6. Chlorophyll Measurement Using SPAD

The chlorophyll content of the leaves was measured using the Konic Minolta SPAD-502 Plus at five different points and its average was considered [36]. Three fully expanded leaves of each plant were considered for the measurements.

### 2.7. Preparation of Leaf Extract

Shoots of the experimental plants were dried to a constant weight in an oven at 40 °C and homogenized into powder. Next, 1 g of the homogenized sample was mixed with 10 mL of 70% ethanol, sonicated in a bath sonicator for 1 h, and centrifuged at 13000× *g* for 15 min. The supernatant was transferred to a fresh tube and tested for various assays [28].

### 2.8. Estimation of Total Reducing Sugar

The anthrone method was followed to estimate the amount of reducing sugars present in the homogenized dry shoot samples [37]. The homogenized plant samples (100 mg) were hydrolyzed with 5 mL of 2.5 N HCl in a boiling water bath for 3 h. The hydrolysate was neutralized with solid sodium carbonate till the effervescence ceased. The volume of the hydrolysate was made up to 100 mL by adding distilled water and centrifuged at 4000 RPM for 10 min, to collect the supernatant. Next, 5 mL of the supernatant was treated with 8 mL of ice-cold anthrone reagent, heated in a boiling water bath for 8 min and the absorbance was recorded at 620 nm after cooling. The concentration of the reducing sugars in the plant samples was determined using the standard curve established with glucose [38].

### 2.9. Antioxidant Activity Assay

The antioxidant activity in the leaf extracts of *Amaranthus hybridus* was determined using the DPPH (diphenyl-picrylhydrazyl) and ABTS (2,2’-azino-bis (3-ethylbenzothiazoline-6-sulfonic acid)) radical degradation technique.

DPPH assay: 10 μL of leaf extract is treated with 1 mL (250 μM) DPPH solution and incubated in dark at room temperature for 30 min. A change in color is estimated by recording the absorbance at 517 nm in a Perkin Elmer Lambda 25 UV/Visible spectrophotometer [39].

ABTS+ assay: Equal amount of ABTS (7.4 mM) and potassium persulfate (2.6 mM) were mixed and incubated at room temperature for 12 h in the dark. ABTS solution and the leaf extracts were mixed in a 19:1 ratio and incubated in the dark for 2 h. The absorbance against methanol was measured at 734 nm using a Perkin Elmer Lambda 25 UV/Visible spectrophotometer [40,41].

The percent inhibition of DPPH and ABTS+ relative to the control was used to determine antioxidant activity using the following equation:

Antioxidant activity (%) = [(Abs. blank − Abs. sample)/Abs. blank] × 100

where Abs. blank—absorbance of the control 10 μL methanol for DPPH and 150 μL methanol for ABTS instead of *Amaranthus hybridus* leaf extract.

Abs. sample is the absorbance of the leaf extracts.

Ascorbic acid was used as a reference standard, and the results obtained are expressed as μg ascorbic acid equivalent g^−1^ (μg AAE g^−1^) dry weight.

### 2.10. Total Phenolic Contents

The modified gallic acid equivalent method was followed for the estimation of phenolic and polyphenolic compounds [42]. The leaf extract was treated with 2 N Folin–Ciocalteu reagent and 7.5% sodium carbonate solution was added. The mixture was incubated in a water bath at 45 °C for 40 min. The absorbance of the mixture was read at 675 nm using a Perkin Elmer Lambda 25 UV/Visible spectrophotometer. Pure gallic acid was used as standard to develop a calibration curve (y = 0.807x + 0.1164 R^2^ = 0.998), which was used to determine the concentration of the phenolic compound as milligrams of gallic acid equivalent per gram of dry weight (mg GAE/g) [42].

### 2.11. Total Flavonoid Contents

Quercetin was considered as the standard sample to compare the total flavonoid contents present in the plant extracts; 5% NaNO_3_ was added to the leaf extract and mixed by vortexing. The mixture was allowed to stand for 6 min, following which, 10% AlCl_3_ was added to the and incubated at room temperature for 5 min. Next, 1 M NaOH was added and the volume was made up to 5 mL with distilled water. The mixture was further incubated at 40 °C for 20 min, after which the absorbance was recorded at 430 nm using a Perkin Elmer Lambda 25 UV/Visible spectrophotometer [43]. The total flavonoid content (TFC) was calculated using the standard quercetin calibration curve, y = 0.605x + 0.0132, R2 = 0.9956 and was expressed in mg QE/g dry extract weight.

### 2.12. Estimation of Cu^2+^ and Zn^2+^ Ions

Root and shoot samples were digested with 5% nitric acid in a microwave-assisted digestor. The digested samples were filtered through grade 40 ashless Whatman filter paper and analyzed for Cu^2+^ and Zn^2+^ ions using Perkin Elmer-Optima 8300 ICP-OES. Three-point calibration curves were established for copper and zinc using commercial ICP standards [44].

### 2.13. Statistical Analysis

All the analyses were performed in triplicates, which were averaged to obtain the replication means and standard deviations. The Shapiro–Wilk (normality of errors) test (*p* > 0.05) was used to determine the normal distribution of the results [45]. Experimental data were statistically evaluated by one-way analysis of variance (ANOVA) using SPSS version 19. A Tukey’s post hoc test was performed to identify the distinct groups that differed from one another across different dependent variables [46]. Comparison between foliar and hydroponics was done using an independent *t*-test [47]. All statistical interpretations were based on a 5% probability (*p* < 0.05).

## 3. Results

The rise in global population and the need for increased agricultural produce are gathering attention. The application of biogenically synthesized metal nanoparticles could overcome this ongoing issue. A multi resistant bacterium, *Stenotrophomonas maltophilia*, isolated from the soil was capable of synthesizing CuO and ZnO nanoparticles in the laboratory media.

### 3.1. Characterization of Biogenic CuO and ZnO Nanoparticles

**Figure 1:**
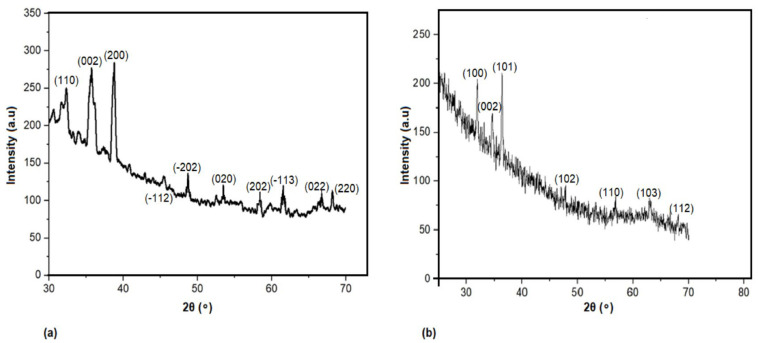
X-ray diffraction (XRD) pattern of (**a**) bacteriogenic CuO nanoparticles and (**b**) bacteriogenic ZnO nanoparticles synthesized by *Stenotrophomonas maltophilia.*

**Figure 2:**
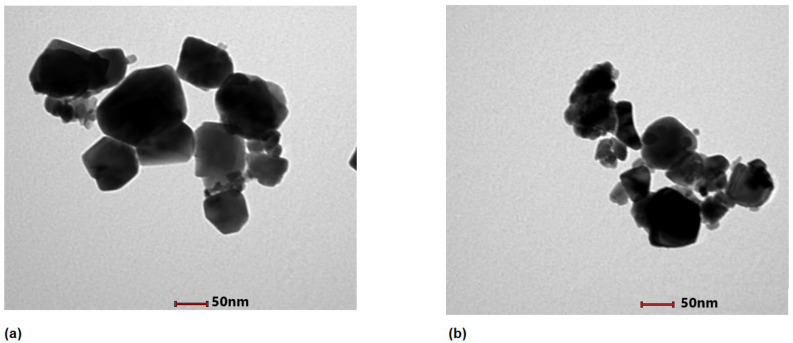
Transmission electron micrographs of (**a**) bacteriogenic CuO nanoparticles and (**b**) bacteriogenic ZnO nanoparticles synthesized by *Stenotrophomonas maltophilia* using JEOL-2100TEM with a scale bar of 50 nm.

**Figure 3:**
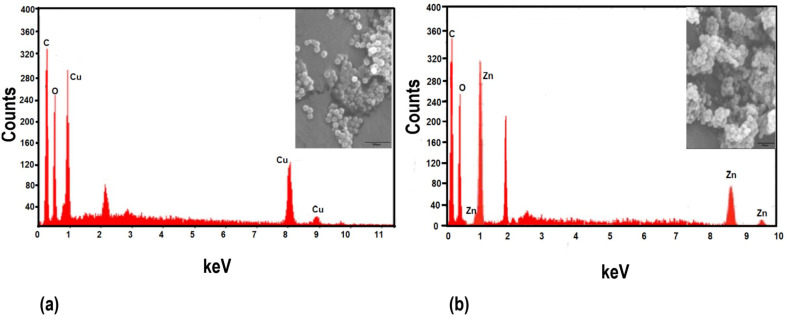
Energy dispersive X-ray diffraction spectrum for (**a**) CuO nanoparticles and (**b**) ZnO nanoparticles. Inset are scanning electron microscopy images of (**a**) bacteriogenic CuO nanoparticles (**b**) bacteriogenic ZnO nanoparticles using JEOL JSM-7600F FEG-SEM with a scale bar of 500 µm.

**Figure 4:**
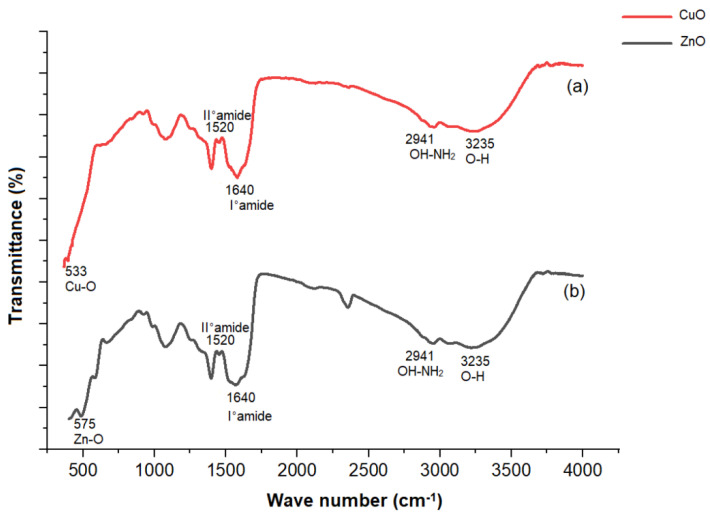
FTIR spectra of (**a**) bacteriogenic CuO nanoparticles and (**b**) bacteriogenic ZnO nanoparticles synthesized by *Stenotrophomonas maltophilia.*

The biogenic synthesis of CuO and ZnO nanoparticles was confirmed by X-ray diffraction (XRD) analysis (Figure 1), TEM image (Figure 2), energy dispersive X-Ray spectroscopy (Figure 3), and FTIR analysis (Figure 4). The XRD peaks observed for the lyophilized nanoparticle samples in Figure 1a correspond to (111), (000), (200), (−112), (−202), (020), (202), (−113), (022), and (220) planes confirming the presence of copper oxide [48], while the 2θ values of 31.94, 34.64.36.42, 47.83,56.85,62.93, and 68.2 (100, 002, 101, 102, 110, and 103,112) observed in Figure 1b can be attributed to the zinc oxide diffraction [32].

The average grain size of the CuO and ZnO was calculated from the full width at half maximum (FWHM) of the diffraction curves by using the Debye–Scherrer formula, [49,50]

$$D=\frac{k\lambda }{\beta \cos\theta }$$

where D is the particle size; K—Scherrer constant (0.9); λ—wavelength of X-ray radiation (0.15406 nm); β—full width at half maximum (FWHM) of the XRD peak; θ—Bragg’s angle.

Using the Debye–Scherrer equation, the average grain size of the CuO and ZnO nanoparticles were determined as 38.96 nm (±5) and 42.64 nm (±3), respectively (Table 1). The particle size for CuO and ZnO nanoparticle ranged between 50 and 100 nm as confirmed by transmission electron microscopy (JEOL-2100TEM) (Figure 2a,b).

Scanning electron microscopic (SEM) analysis revealed the spherical morphology of CuO and ZnO nanoparticles and confirmed the size of less than 100 nm (Figure 3 inset). The energy dispersive X-ray spectroscopy (Figure 3) clearly indicates the presence of Cu and Zn in the respective nanoparticles, along with O.

In Figure 4a, the FTIR peaks at 533 cm^−1^ correspond to the typical stretching vibration of the Cu–O bond, while in Figure 4b, the FTIR peaks at 575 cm^−1^ relate to Zn–O stretching. The appearance of bands with wavenumbers of 1640 cm^−1^ and 1520 cm^−1^ corresponds to the bending vibrations of the protein amides I and II, respectively. The peaks around 2941 cm^−1^ and 3235 cm^−1^ are attributed to the stretching vibrations of amide I superimposed on the side of the hydroxyl group band [51,52], which supports the existence of protein and, consequently, the biosynthesis of CuO and ZnO nanoparticles.

### 3.2. Seed Priming

**Figure 5:**
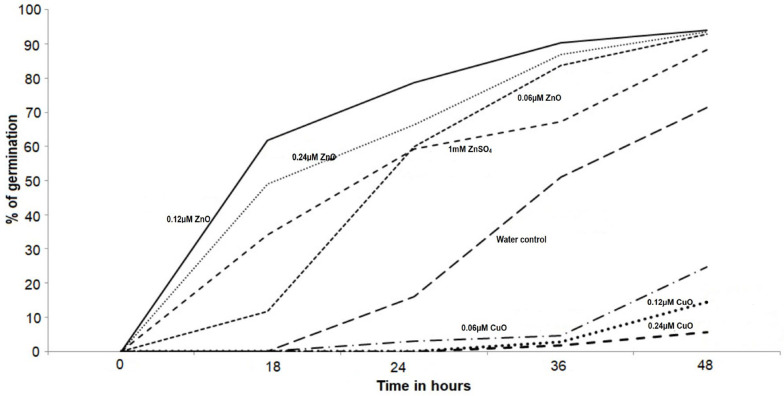
Seed priming of *Amaranthus hybridus* seeds with varying concentrations of CuO and ZnO nanoparticles to determine their optimal concentration for seed germination. A water control was maintained along with zinc salt control (1 mM ZnSO4).

ZnO nanoparticles applied to *Amaranthus hybridus* seeds demonstrated expected results (Figure 5) with increased percent seed germination, as compared to water control and 1 mM ZnSO_4_ salt solution. It is evident from Figure 5 that the percent seed germination was initially dependent on the concentration of ZnO nanoparticles, however, after 48 h, all the selected concentrations of ZnO nanoparticles demonstrated a similar effect, resulting in more than 90% seed germination. A significant variation was observed between the percent seed germination for seeds primed with water control and 0.06, 0.12, and 0.24 µm ZnO nanoparticles, resulting in an increase of 33.7%, 61.7%, and 49% seed germination, respectively. It was heartening to observe a significant increase in percent germination from 71.33% for water control to above 90% for ZnO-primed seeds. Additionally, 1 mM ZnSO_4_ salt solution used as control for zinc also demonstrated an increase in percent germination of up to 86.5%, but failed to cross the 90% mark. An exact reverse effect was observed with CuO nanoparticles, where percent seed germination decreased with increased concentration of CuO nanoparticles (Figure 5). Excess copper in soil is reported to inhibit the growth of roots prior to leaf development and affect metabolic processes in the plant [53,54,55]. Figure 4 clearly exhibits a negative influence of CuO nanoparticles, where the percent germination of seeds decreases from 71.33% for water control to 24.6%, 14.3%, and 5.7% germination, with increased CuO concentrations at 0.06, 0.12 and 0.24 µm, respectively.

### 3.3. Optimal Concentration of CuO and ZnO Nanoparticles

**Figure 6:**
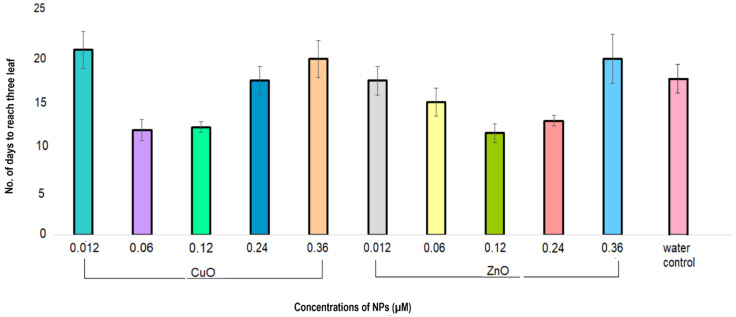
Effect of varying concentrations of CuO and ZnO nanoparticles on the development of *Amaranthus hybridus* seeds to reach the three-leaf stage. CuO and ZnO nanoparticles in the concentrations 0.012, 0.06, 0.12, 0.24, and 0.36 µm were spiked in half-strength Hoagland’s medium. A water control and media controls were maintained. Bars represent the mean and standard error (SE) of three replicates (three seeds in each replicate). Significant differences between the means were calculated at *p* < 0.05.

The seeds germinated in DI water were transferred to half-strength modified Hoagland’s medium for growth. CuSO_4_ and ZnSO_4_ salts in the Hoagland’s medium were replaced with varying concentrations of CuO and ZnO nanoparticles. Plantlets grown in concentrations of 0.06 µm, 0.12 µm for CuO and 0.12 µm, and 0.24 µm for ZnO took significantly less time to attain the three-leaf stage as compared to the water control (Figure 6). The plantlets grown in the presence of 0.06 µm CuO and 0.12 µm ZnO nanoparticles reached the three-leaf stage in 11 days, with an approximate 38% reduction in time as compared to the water control, which took 17 days to attain the same development stage. The next effective concentrations of CuO and ZnO nanoparticles were 0.12 µm and 0.24 µm, respectively, which took 12 and 13 days, respectively, to attain the three-leaf stage. Since these four concentrations of nanoparticles exhibited a significantly faster development of the plantlets as compared to water control, they were selected for further studies using hydroponic and foliar systems.

### 3.4. Growth of the Plants in Pot Study and Hydroponic System

**Figure 7:**
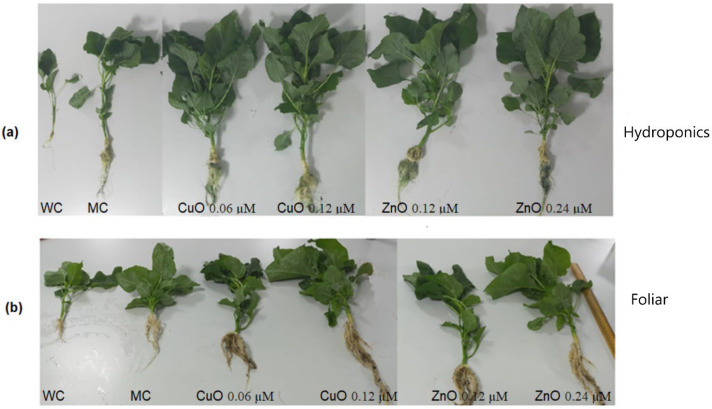
Phenotypic representation of *Amaranthus hybridus* plants treated with varying concentrations of CuO and ZnO nanoparticles (0.06 μM CuO, 0.12 μM CuO, 0.12 μM ZnO, and 0.24 μM ZnO) (**a**) in hydroponics setup and (**b**) foliar application. A water control (WC) and media control (MC) were maintained.

The plantlets grown till the three-leaf stage were transferred to the hydroponic system and soil pots for further study. Both systems were treated with the selected four concentrations of the nanoparticles and the growth parameters, such as leaf number, leaf surface area, total length of the plant, wet mass, and dry mass were monitored for 60 days. Water and media controls were maintained to compare and analyze the difference in plant growth. The results of the application of CuO and ZnO nanoparticles through hydroponic and foliar systems on plantlets of *Amaranthus hybridus* are presented in Table 2. The application of CuO and ZnO nanoparticles on the *Amaranthus hybridus* plants grown in both systems demonstrated a positive effect. The phenotypic image of nanoparticle-treated plants in Figure 7 clearly evinces the effect.

#### 3.4.1. Foliar Application of Fertilizers

One-way ANOVA and Tukey’s post hoc analysis displayed a statistically significant increase in numbers of leaves, leaf surface area, plant length, as well as total wet and dry mass for all the four nanoparticle treatments as compared with water and media controls (Table 2) (*p* < 0.05). The maximum number of leaves (32.3) observed in plants treated with 0.24 µm ZnO nanoparticles optimally supported the plant growth (Table 2). All the growth parameters expressed the best values with 0.24 µm ZnO nanoparticles except the leaf surface area, which was insignificantly better with 0.12 µm ZnO nanoparticles. In this study, a comparison between CuO and ZnO nanoparticles reveals a clear vision, suggesting ZnO nanoparticles as a better choice, though CuO are also efficient as compared to the media and water controls.

#### 3.4.2. Hydroponics Application of Fertilizer

Similar to the foliar application, hydroponic systems also revealed 0.24 µm ZnO nanoparticles as best for all growth parameters, except number of leaves, where 0.12 µm ZnO nanoparticles were insignificantly better (Table 2). The highest number of leaves (42 ± 1.8) were found in plants treated with 0.12 µm ZnO nanoparticles, while enhanced leaf surface area (78 ± 3.6), plant height (63.1 ± 3.7), fresh weight (66.1 ± 4.7), and dry weight (8.6 ± 0.89) were noted in plants treated with 0.24 µm ZnO.

### 3.5. Chlorophyll Measurement in Plants Treated with CuO and ZnO Nanoparticles

**Figure 8:**
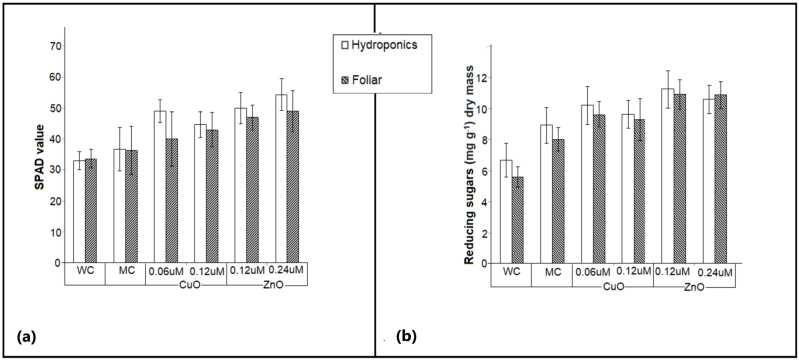
Effect of varying concentrations of biogenic CuO and ZnO nanoparticles applied under hydroponic and foliar setup on biochemical parameters (**a**) SPAD value, (**b**) reducing sugar content in the *Amaranthus hybridus* plant. A water control (WC) and media control (MC) were maintained.

The SPAD values of the *Amaranthus hybridus* plants grown in foliar and hydroponic systems were recorded. These values have a direct correlation with the amount of chlorophyll in the plant leaves and are therefore an index for the plant growth [56]. All the plants treated with CuO and ZnO nanoparticles demonstrated a significantly higher (*p*-value > 0.05) SPAD value as compared with those observed for water and media controls (Figure 8a). The highest SPAD value 54.5 ± 5 was measured for 0.24 µm ZnO nanoparticles in the hydroponic system followed by 49 ± 3 for 0.24 µm ZnO nanoparticles for foliar application (Figure 8a). This insignificant difference between the SPAD values of the plants under different treatments suggests the influence of ZnO nanoparticles in plants and an at-par uptake of zinc through both systems. Zinc ions form a structural and catalytic component of proteins and enzymes, as well as a co-factor for the normal development of pigment biosynthesis, and their deficiency critically impacts chlorophyll concentration in the leaves [57]. Increased zinc ions improve the photosynthetic efficiency of the plant [58,59]. Similarly, the lower amount of copper ions also enhances chlorophyll synthesis, however, an increased amount could be toxic to the photosynthetic apparatus [60,61]. This is clearly demonstrated in Figure 8a, where the SPAD value is seen to marginally decrease for 0.12 µm CuO as compared with 0.06 µm CuO.

### 3.6. Estimation of Reducing Sugar in Plants Treated with CuO and ZnO Nanoparticles

The total reducing sugar in plants treated with CuO and ZnO nanoparticles was determined using the calibration curve (y = 1.0015x + 0.049 R^2^ = 0.984) for standard glucose and was reported as mg/g dry mass. All the plants treated with nanoparticles demonstrated a significantly higher (*p*-value > 0.05) reducing of sugar content, as compared with those observed for water control (Figure 8b). The experimental plants exhibited a similar pattern to the growth parameters and SPAD values, where ZnO nanoparticles proved to enhance the reducing sugar content in the plants. It was found that 0.12 µm ZnO nanoparticles accumulated marginally more reducing sugars as compared with other treatments. The addition of copper and zinc in the nutrient medium increased the photosynthetic efficiency due to increase in chlorophyll pigment resulting in a significant increase in the synthesis of reducing sugars [62].

### 3.7. Antioxidant Activity

**Figure 9:**
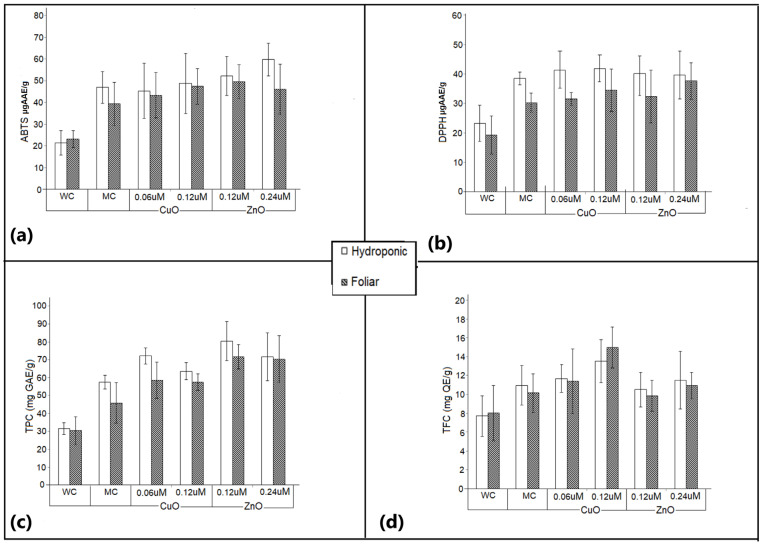
Effect of varying concentrations of biogenic CuO and ZnO nanoparticles applied under hydroponic and foliar setup on antioxidant activity measured by (**a**) ABTS assay, (**b**) DPPH assay, and (**c**) total phenolic content. (**d**) Total flavonoid content in *Amaranthus hybridus* plant. Significant difference (*p* < 0.05). A water control (WC) and media control (MC) were maintained.

Foliar application of ZnO nanoparticles increased the gene expression of antioxidative enzymes, transporters, and the enzymes involved in metabolism of Fe-deficient tomato plants [58]. The level of antioxidant activity in the experimental plants performed by the ABTS assay is shown in Figure 9a. It was evident that the measured antioxidant activity was different for the various treatments in both the setups. The antioxidant activity ranged significantly from 21.34 µg AAE/g for hydroponic water control, to 59.74 µg AAE/g for 0.24 µm ZnO nanoparticle-treated plants in the hydroponic system. CuO nanoparticle treatment did not exhibit a significant difference from the media control under the hydroponic system, but plants treated with 0.12 µm CuO nanoparticles under foliar application expressed a significant variation (*p* > 0.05). ZnO nanoparticles encouraged a significantly higher antioxidant activity under both hydroponic and foliar setups, as compared to media controls recording 49.55 µg AAE/g for 0.12 µm ZnO.

The antioxidant activity measured by the DPPH assay did not express a strong variation between the different nanoparticle treatments (Figure 9b). Under foliar application, the activity observed for media control, 0.06 µm CuO nanoparticles and 0.12 µm ZnO nanoparticles did not express a significant variation. Plants treated with 0.24 µm ZnO nanoparticles had the highest activity. However, the assay failed to bring out significant differences in the antioxidant activity between the nanoparticle-treated plants. The antioxidant activity was significantly higher in nanoparticle-treated plants, as compared with water control, in both foliar and hydronics. The effect of antioxidants on DPPH radical scavenging is a result of their ability to donate hydrogen or radical scavenging action.

### 3.8. Total Phenolic Content

Assaying the total phenolic content is a method for quantifying the antioxidant potential. The Folin–Ciocalteu method was followed for the quantitative determination of the total phenolic content. The total phenolic content (TPC) of the experimental plants ranged from 31.56 ± 3.27 to 80.39 ± 9.21 mg GAE/g dry extract weight (Figure 9c). TPC content was significantly higher in the nanoparticle-treated plants than in both the water and media control for foliar treatment; however, for plants grown in hydroponic systems, the water control exhibited significantly lower TPC, as compared with the nanoparticle-treated plants. Among all the samples, maximum TPC was observed in plants treated with 0.12 µm ZnO nanoparticles, with 80.39 ± 9.21 mg GAE/g for hydroponic system and 71.66± 6.85 mg GAE/g for foliar treatment. The lowest TPC (30.75 ± 7.8 mg GAE/g) was observed in water control plants under foliar treatment. An approximate 62% increase in TPC is observed due to 0.12 µm ZnO nanoparticles, as compared with water control. It is interesting to note a higher TPC among the plants treated with lower concentrations of the CuO and ZnO nanoparticles, signifying a negative influence of CuO and ZnO at higher concentrations.

### 3.9. Total Flavonoid Content

TFC estimation revealed an increase in flavonoid content in extracts of *Amaranthus hybridus* plants treated with CuO and ZnO nanoparticles. The 0.12 µm CuO nanoparticles supported maximum flavonoid content (15.44 ± 3.42 mg QE/g) in plants under foliar application followed by the hydroponic system (13.77(±3.71 mg QE/g) (Figure 9d).

**Figure 10:**
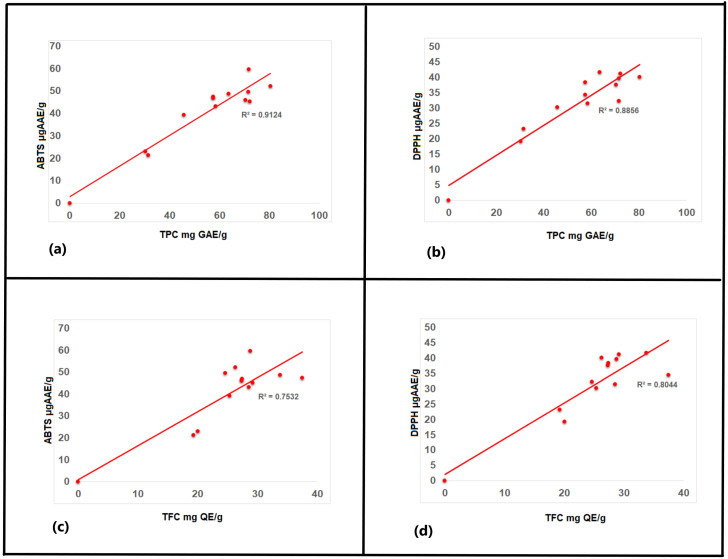
Correlations between the antioxidant activity, total phenolic content, and total flavonoid content in *Amaranthus hybridus.* (**a**) TPC *v*/*s* ABTS, (**b**) TPC *v*/*s* DPPH, (**c**) TFC *v*/*s* ABTS, and (**d**) TFC *v*/*s* DPPH.

A significant positive correlation was observed between the antioxidant activity of the ABTS and DPPH assay (r = 0.946). The correlation data between the ABTS/DPPH assay and the total phenolic content were r = 0.955 and r = 0.941 for ABTS and DPPH, respectively (Figure 10), while the correlation between the TFC and ABTS/DPPH assay was r = 0.645 and r = 0.626 for ABTS and DPPH, respectively. Similar results were reported for different species of Amaranthus [63]. This stipulates the influence of TPC and TFC on the antioxidant capacity of the plants. A positive correlation (r = 0.829) is observed between TPC and TFC, indicating flavonoids as one of the major constituents of TPC.

### 3.10. Uptake of Copper and Zinc in Plant Tissue

**Figure 11:**
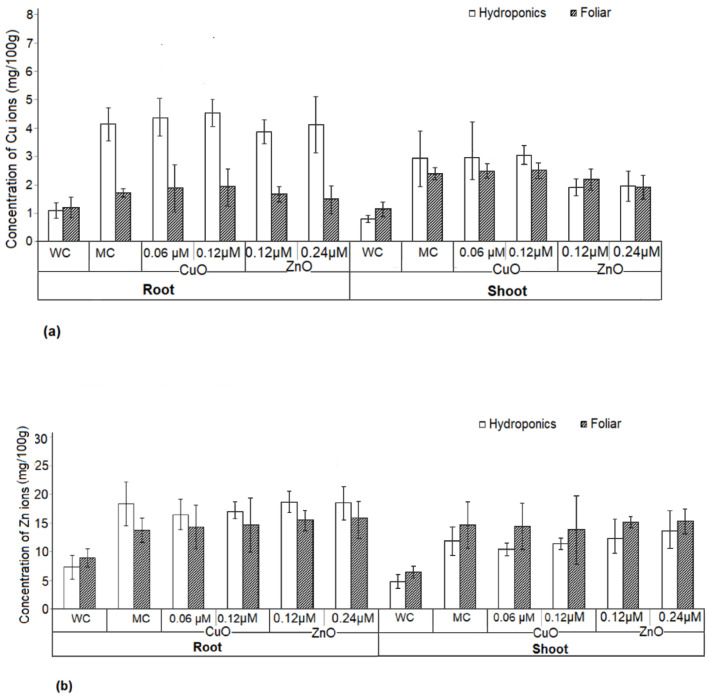
Uptake of (**a**) copper and (**b**) zinc in the roots and shoots of *Amaranthus hybridus* treated with varying concentrations of CuO and ZnO nanoparticles under hydroponics and foliar setups. A water control (WC) and media control (MC) were maintained. Significant difference (*p* < 0.05).

The copper and zinc concentrations in shoot and root were estimated for both hydroponic- and foliar-treated plants to understand if the treatment method has any effect on mineral allocation in the plant system (Figure 11). The total copper and zinc content in the shoots and roots of *Amaranthus hybridus* plants significantly increased in the nanoparticle treatments in both foliar and hydroponics, as compared with water control. Copper accumulation was significantly higher in the roots of plants under the hydroponic system when compared with water control. However, no significant difference was observed in the concentration of copper and zinc in the root and shoot of the plants treated with nanoparticles and media control. This suggests no accumulation of copper and zinc due to CuO and ZnO nanoparticle application.

## 4. Discussion

The current investigation demonstrated the significant influence of the biogenic CuO and ZnO nanoparticles on all the biochemical variables and most of the morphological traits of *Amaranthus hybridus.* ZnO nanoparticles were reported to increase the vigor of seed germination and plant growth [64]. Salam et al. (2022) reported the positive influence of ZnO nanoparticles in growth of maize plant when the seeds were primed with a 500 mg/L concentration of ZnO nanoparticles [65]. Zinc is vital in seed germination, plant growth, and development, and is involved in the synthesis of protein and plant hormones (auxin), cell growth, and in stress resistance. The biogenic ZnO nanoparticles were found to improve the seed germination rate by 39%, whereas CuO nanoparticles exert a counter effect. Priming allows seeds to sprout and emerge even in harsh agro-climatic circumstances, such as cold and damp or extreme heat [66]. Uniform germination assists in optimizing harvesting efficiency, which can boost production potential. If all seeds sprout simultaneously, it is likely that they develop into plants that will be ready for harvest at the same time [67]. The contribution of zinc in seed priming is not completely understood, however, reports indicate their role pin the initial stages of radicle and coleoptile development [68]. Both CuO and ZnO nanoparticle-treated *Amaranthus hybridus* plants showed improved growth parameters, photosynthesis, and antioxidant activities, but ZnO nanoparticle treatment had a better influence. ZnO nanoparticles exert a beneficial effect on auxin production, which stimulates cell division and mineral absorption, hence promoting plant development [69,70], while reducing oxidative stress [70] or environmental stress [57,59]. Zinc deficiency, hence, affects the quality of the crop yield, as well as the crop product [71,72]. Sun et al. (2020) used the foliar application of ZnO nanoparticles to demonstrate their critical role in overcoming iron deficiency in tomato plants [58]. Similarly, Timothy et al. (2003) applied zinc in hydroponic systems to study their effect on the growth of Brassica plants [73]. ZnO nanoparticles also influence the uptake of other metals, such as copper, manganese, and iron in plants, thereby affecting the plant growth [74]. The application of copper nanoparticles is reported to boost the growth of basil, rice, wheat, maize, and tomatoes [28,75,76]. Copper plays a vital role in the production of regulatory proteins, as well as in mitochondrial respiration and hormone signaling, which finally results in the enhancement of plant growth parameters [77]. Shende et al., 2016 [78] studied the role of copper nanoparticles in enhancing the growth of pigeon pea plants using the conventional method of fertilizer application in soil for copper nanoparticles, which resulted in larger quantities (20 ppm) of copper nanoparticles being applied. In the current study, however, nanoparticles are applied at much lower concentrations (0.24 µm), which highlights the benefits of foliar application and hydroponic systems over conventional farming techniques.

SPAD values are directly associated with pigment concentration and are a valid indicator of chlorophyll content in plant leaves. Both ZnO and CuO nanoparticles are seen to support chlorophyll synthesis, thereby promoting plant growth. However, CuO at higher concentrations is observed to exert a damaging effect on the chlorophyll content. Previous reports on deficiency of copper and zinc demonstrated reduction in chlorophyll content, which appears as chlorotic symptoms, and results in a significant reduction in plant growth [79]. Higher SPAD values can be correlated with enhanced photosynthetic activity and carbon assimilation. This is reflected as the concentration of reducing sugars in the plant biomass. A direct correlation is observed between the photosynthetic activity and the concentration of reducing sugars. Reports in the literature confirmed an inverse correlation between the chlorophyll content and starch content in the *Vigna radiata* and okra plants treated with ZnO nanoparticles [59,80]. Similar observations are also reported in *Tetraselmis* sp. [81]. The rate of metabolism rises when plants have access to nutrients or any fertilizer [82]. Application of ZnO and CuO nanoparticles, hence, aids in improving the photosynthetic activity of the plant.

The exposure of plants to various types of stress, biotic or abiotic, leads to overproduction of reactive oxygen species (ROS), which are powerful oxidizing agents and toxic to cells [68]. The synthesis and accumulation of many antioxidants, including phenolic acids, flavonoids, vitamin C, carotenoids, glutathione, etc., scavenge ROS and act as a natural defense mechanism for plants against stress [83,84,85,86,87]. Moreover, they also are antimicrobial and, therefore, effective against fungal and bacterial infections and plant pests [88]. In the current study, ZnO nanoparticle treatment reported higher antioxidant activity in plants by the ABTS assay in both hydroponic and foliar application systems, as compared with media and water controls, while CuO treatment on plants under the hydroponic system did not demonstrate any significant variation in media control but was effective on plants under foliar treatment. The DPHH assay failed to reveal any significant difference in the antioxidant activity between the nanoparticle-treated plants, though the activity was significantly higher as compared with water and media controls. Thus, an increased antioxidant activity among the nanoparticle-treated plants signifies better tolerance towards infections and pests [88,89]. Similar reports are published in rice, cauliflower, and tomato after copper and cabbage, cauliflower, tomato, black mustard, and pepper after zinc fertilization, respectively [28]. Amongst the two assays tried for the antioxidant activity, ABTS expressed a higher level of significance as compared to the DPPH assay. Floegel et al. (2011) reported a similar finding while comparing the ABTS and DPPH assays to measure the antioxidant capacity in foods [90]. There are many reports on the use of nanoparticles stimulating the formation of various types of antioxidants, such as phenolic compounds. Flavonoids are the largest group of phenolic compounds that contribute to the organoleptic properties, such as the color, smell, and taste of fruit and flowers, and also protect from pest attack [84,88,89]. Zinc and copper play an important role in the expression of the phenolic pathway and synthesis of phenolic compounds [91,92,93]. The experimental results of *Amaranthus hybridus* plants treated with ZnO and CuO nanoparticles display a higher TPC and TFC, respectively. It is interesting to note a lowered TPC at higher concentrations of CuO and ZnO nanoparticles. There are a few reports of excess copper ions stimulating the synthesis of flavonoids, specifically luteolin glycosides, in the absence of UV light [94], while Kachel et al., in 2022, reported the negative influence of copper nanoparticles on the TFC of seeds [88]. Thus, application of CuO and ZnO nanoparticles encourages production of antioxidants, which at a given time and less lethal dosage can be advantageous for increasing plant’s resistance to infections and enhances the plant growth [95], which is corroborated with the current study.

According to reports, ions from CuO and ZnO nanoparticles dissociate slowly, making the ions readily available for the plant roots to absorb in a hydroponic system [96]. The study confirmed that there was no increased intake of copper and zinc minerals, as the readings were at par with media control. *Amaranthus hybridus* plants are consumed for its leaves, and hence, the accumulation of copper and zinc in the shoot can raise concern. The recommended dietary allowance (RDA) for copper and zinc is 900 µg and 8–11 mg, respectively [97,98], hence the amount of copper and zinc in the shoot of the nanoparticle-treated plants is much lower (2.93 mg/100 g dry weight and 14.72 mg/100 g dry weight) than the RDA by the National Institute of Health (NIH).

Toxicity of CuO and ZnO nanoparticles above 1000 mg/L is reported in *Raphanus sativus* and *Spinacia oleracea,* which resulted in reduced shoot and root length [30,99]. Hence, it is important to note the significance of nanoparticle concentration, where increased dosage results in toxic effects. In the current study, the concentration of CuO and ZnO nanoparticles is used at a much lower range, i.e., a nanoscale, and hence, there was less or no phytotoxicity observed.

## 5. Conclusions

The outcomes of this study demonstrate the efficient use of green synthesized CuO and ZnO nanoparticles as nanofertilizers by enhancing the agronomic features of *Amaranthus hybridus* in both hydroponics and foliar application. The independent sample *t*-test *p* ≤ 0.05 showed that the plants under hydroponic application of nanoparticles had a statistically significant higher mean of agronomic parameters, synthesis of antioxidants, as well as accumulation of copper and zinc ions in comparison to the foliar application plants. This could be attributed to the easy uptake of nutrients through the roots in the plants under the hydroponic setup. The small size of the nanoparticles offers better efficiency with respect to penetration and transport. This resulted in use of almost 70% lower concentrations of CuO and ZnO nanoparticles than the CuSO_4_ and ZnSO_4_ salts in the nutrient media. Many researchers unveiled the potential of CuO and ZnO nanoparticles in agriculture, but the biogenic nanoparticles produced by *S. maltophilia* possessed a higher efficiency and could demonstrate the same results at lower concentrations in the nano range. This is beneficial in preventing their accumulation in soils and thereby avoiding environmental pollution. Nanoparticle-treated plants of *Amaranthus hybridus* exhibited improved growth as compared to the plants grown in Hoagland’s media and water control. No phytotoxic symptoms were observed throughout the study, which could be attributed to the micro molar concentration of the nanoparticles, which are much lower than the toxic levels reported for plants. The present study concludes that biogenic nanoparticles can be used as efficient fertilizers, resulting in higher quality and quantity yield while protecting the environment. The study thus could be used to solve the ongoing issue of food crop shortage and can provide basic information for developing a nutrient medium composed of micro and macro nanoparticle elements.

## Figures and Tables

**Table 1:** Determination of the crystallite size of biosynthesized CuO and ZnO nanoparticle synthesized using XRD analysis.

	Peak Position (2θ)	FWHM (θ)	Crystallite Size (nm)	Average Crystallite Size (nm)
CuO	20.90853	0.22918	35.24957	38.96 (±5)
	35.37912	0.27172	30.68807	
	38.61757	0.1803	46.68871	
	48.62369	0.27196	32.05383	
	61.41574	0.25925	35.64097	
	66.0172	0.17732	53.42576	
ZnO	23.28048	0.26135	31.03541	42.64 (±3)
	31.8997	0.24101	34.28227	
	33.47986	0.21567	38.46548	
	34.76525	0.17377	47.90504	
	50.91306	0.14305	61.50663	

**Table 2:** Effect of the application of CuO and ZnO nanoparticles through hydroponic and foliar systems on the phenotypic character of *Amaranthus hybridus* plantlets.

	Foliar						Hydroponics					
	CuO 0.06 µm	CuO 0.12 µm	ZnO 0.12 µm	ZnO 0.24 µm	Water Control	Media Control	CuO 0.06 µm	CuO 0.12 µm	ZnO 0.12 µm	ZnO 0.24 µm	Water Control	Media Control
No: of leaves	26	28	27	**32.3**	15.5	13.5	31.3	41	**42**	38	15.3	14.3
Leaf surface-area (cm^2^)	62.5	59.2	**69.2**	67.5	39.7	38.4	63	67.8	72.7	**78**	51	36
Total length (cm)	50.4	51.7	44.4	**62.1**	40	36	56.8	58.2	58.7	**63.1**	41.7	40
Total fresh weight (g)	31	30.8	27.4	**40.3**	14	7.45	43.3	54	51.2	**66.1**	22	13.7
Total dry weight (g)	3.8	3.9	3.6	**5.4**	1.7	0.9	5.5	7.1	6.7	**8.6**	2.8	1.8

## Data Availability

Date is contained within the article.
